# Phylogenomic analysis of the genus *Delftia* reveals distinct major lineages with ecological specializations

**DOI:** 10.1099/mgen.0.000864

**Published:** 2022-09-15

**Authors:** Supriya V. Bhat, Heather Maughan, Andrew D. S. Cameron, Christopher K. Yost

**Affiliations:** ^1^​ Department of Biology, University of Regina, Regina, SK, Canada; ^2^​ Institute for Microbial Systems and Society, University of Regina, Regina, SK, Canada; ^3^​ Ronin Institute, Montclair, NJ, USA

**Keywords:** *Delftia*, gold metabolism, plant-growth promotion

## Abstract

*
Delftia* is a diverse betaproteobacterial genus with many strains having agricultural and industrial relevance, including plant-growth promotion, bioremediation of hydrocarbon-contaminated soils, and heavy metal immobilization. *Delftia* spp. are broadly distributed in the environment, and have been isolated from plant hosts as well as healthy and diseased animal hosts, yet the genetic basis of this ecological versatility has not been characterized. Here, we present a phylogenomic comparison of published *Delftia* genomes and show that the genus is divided into two well-supported clades: one ‘* Delftia acidovorans *’ clade with isolates from soils and plant rhizospheres, and a second ‘* Delftia lacustris* and *Delftia tsuruhatensis *’ clade with isolates from humans and sludge. The pan-genome inferred from 61 *Delftia* genomes contained over 28 000 genes, of which only 884 were found in all genomes. Analysis of industrially relevant functions highlighted the ecological versatility of *Delftia* and supported their role as generalists.

## Data Summary

The genomes that were used are listed in Table S1 (available with the online version of this article) and the data are publicly available through the patric (Pathosystems Resource Integration Center) database.

**Impact Statement**

Bacteria in the genus *Delftia* are notable members of soil communities due to their metabolic capabilities for applied microbiology, such as promoting plant growth and bioremediation. In this article, comparisons of *Delftia* genome sequences indicate two closely related strain groups, each of which has adapted to live in a particular environment: members of the *Delftia acidovorans* clade were closely associated with plants and soil, whereas members of the *Delftia lacustris* and *Delftia tsuruhatensis* clade were associated with industrial sludge or humans. The evolutionary framework offered in this paper will provide researchers with context when identifying strains for further biotechnological development.

## Introduction

*Delftia* are motile, aerobic, non-fermenting, Gram-negative rods in the phylum *Pseudomonadota *, and class of *Betaproteobacteria *. Strains have been isolated from diverse niches, including polluted soil [1–3], wastewater [4], plant rhizosphere soil [5], plant endosphere [6, 7] and from clinical specimens [4, 8–11], highlighting the metabolic capabilities and saprophytic lifestyle of *Delftia *. *Delftia* isolates have demonstrated nitrogen-fixing capabilities as free-living diazotrophs [12, 13], while others are noted as proficient root symbiotic colonizers [6, 14]. Consequently, *Delftia* spp. have attracted attention from the research community as a promising resource for isolation of effective plant-growth-promoting rhizobacteria (PGPR), and bioremediation applications. Specific strains of *Delftia* have been used as agricultural inoculants [15], bioremediation agents [16–18] and for mineralizing metals [19–21]. The intersection between bioremediation capacity and plant-growth promotion is notable where heavy-metal-resistant *Delftia* strains isolated from lead- or chromium-contaminated soil produced siderophores and promoted plant growth [1, 17].

The majority of studies with *Delftia* have focused on specific strains or are restricted to individual species. A recent study of the *Delftia tsuruhatensis* pan-genome showed the extensive gene diversity in this species, highlighting the role of horizontal gene transfer and potential for biotechnological applications [22]. It remains to be determined whether the ecosystem services and potential biotechnological applications provided by individual *Delftia* isolates are widespread throughout the genus or restricted to a few strains or a clade. The broad accessibility to genome sequencing technology has provided the opportunity to explore this question by accessing the publicly available genome sequences of *Delftia* spp. To investigate the ubiquity of plant-growth-promoting and metal-metabolism traits in *Delftia *, we inferred a *Delftia* phylogeny and used it to examine the distributions of groups of genes underlying these ecologically and industrially relevant traits.

## Methods

### Data acquisition

The genomes of 61 *Delftia* isolates were analysed within the Pathosystems Resource Integration Center (patric) 3.6.12 resource [23]. Genomes were included in the analyses if they were considered of ‘good’ quality and had fewer than 500 contigs. Table S1 provides the list of strains, accession numbers and other relevant metadata.

### Pan-genome analysis and shared protein families

Core genomes and pan-genomes were identified using Roary [24] and GNU Parallel [25], with genome input files annotated using prokka [26]. Pan-genomes were evaluated as open or closed using power law regression in R to estimate the gamma parameter [27]. Proteins unique to, or shared between, genomes or certain clades were identified using the Protein Family Sorter of patric, as were distributions of proteins involved in phenotypes of interest, such as root colonization and metal metabolism. Virulence genes were identified by using blastp (*E* value cut-off=1×10^−20^) [28] to query patric predicted proteins from each genome against a local database comprising Virulence Factor Database (VFDB) set A proteins.

### Average nucleotide identity (ANI)

ANI analysis was conducted on all 61 genomes using ANIb within Pyani [29]. ANIb aligns long (1020 nt) segments of coding regions using blastn+ then measures the nucleotide identity between the aligned regions and sums these local alignments across each whole genome [29]. The cluster diagram was produced by Pyani and the distribution of ANI values by clade was plotted in R [30] using ggplot2 [31].

### Phylogenetic analysis

A phylogenetic tree was inferred in patric using the Phylogenetic Tree Building function, with a maximum of five deletions or five duplications, and visualized using FigTree v1.4.4 [32]. Ancestral habitats were inferred using the Trace Character History function in Mesquite [33]. Population structure was assessed using hierBAPS with default parameters in R [34, 35]. Only one genome was included for groups of closely related genomes (>99.98 % ANI) from the same study (* Delftia* sp. S65, *Delftia* sp. S66 and *Delftia* sp. S67 [36]; *Delftia* sp. SD018 and *Delftia* sp. SD083 [37]); for example, only *Delftia* sp. S67 and *Delftia* sp. SD018 were included. Habitat of isolation was considered to evolve according to an unordered model, which allows switching between any habitat without cost.

## Results and Discussion

### Phylogenetic analysis reveals that species within *Delftia* form two clades

**Fig. 1.**
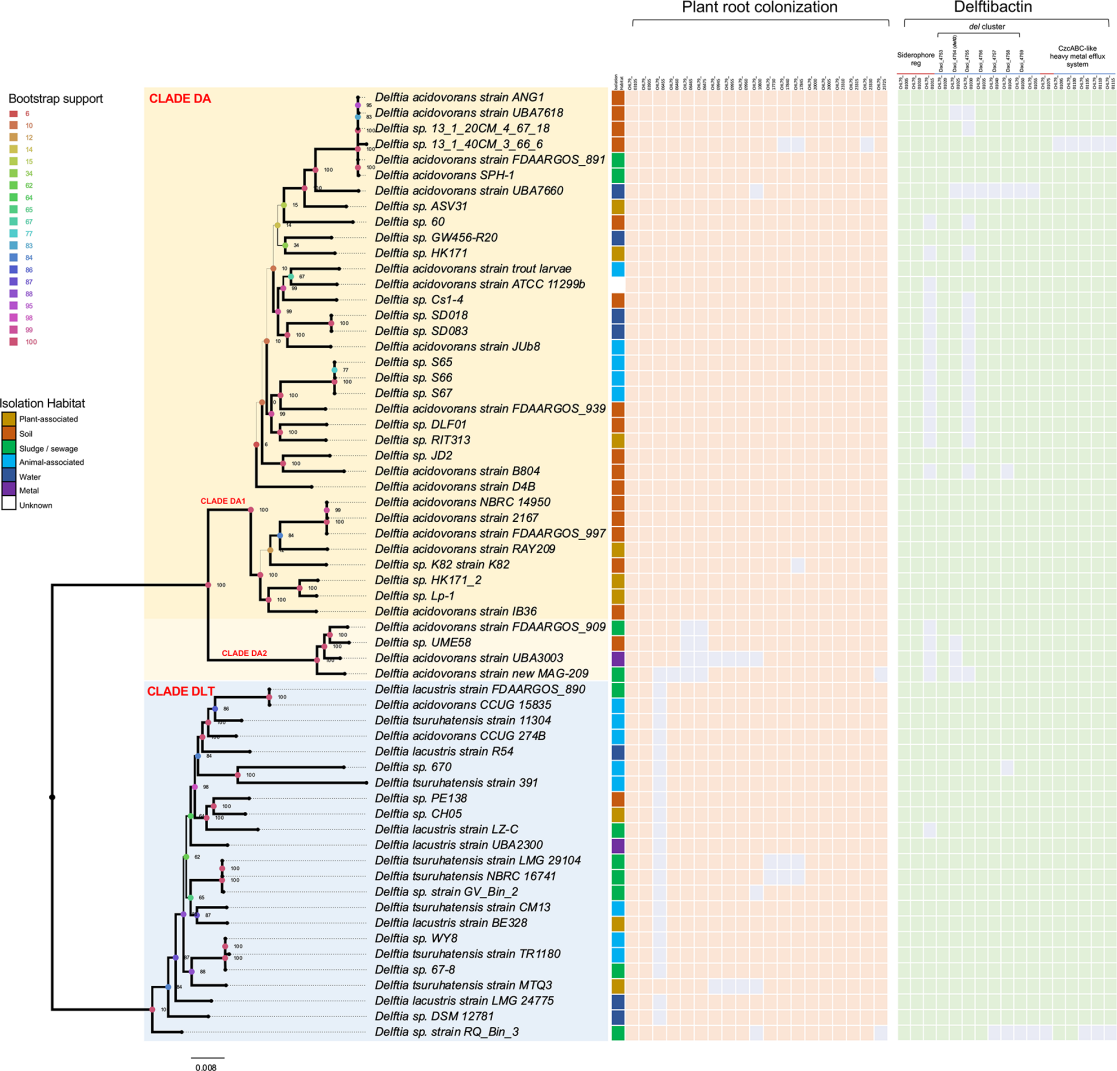
*
Delftia* phylogeny showing two main clades, DA and DLT, and the DA sub-clades, DA1 and DA2. Support values from 1000 bootstrap pseudoreplicates are shown as numbers and as circles coloured according to the ‘bootstrap support’ legend on the left. Branch thickness also indicates support level with thinner branches having lower support. The scale bar at the bottom indicates branch length as the number of substitutions per site. The boxes to the right of the phylogeny show, from the left, (i) habitat of isolation for each strain (colour according to the key on the left), (ii) presence (peach) or absence (grey) of genes shown to be expressed in RAY209 during plant root colonization [49], and (iii) presence (green) or absence (grey) of genes that encode or regulate production of delftibactin and the CzcABC-like heavy metal efflux system.

Sixty-one *Delftia* genomes were selected for phylogenetic analysis based on genome quality and number of contigs (see Methods) (Fig. 1, Table S1). The median genome size was 6.6 Mbp, with a largest size of 7.3 Mbp for *Delftia lacustris* strain FDAARGOS 890 isolated from a sewage treatment plant in Germany [38]. The 61 genomes represented three described species, *Delftia acidovorans* [39], *D. tsuruhatensis* [40] and *D. lacustris* [41], and an additional 27 strains that are not yet classified to the species level. Three additional *Delftia* species are recognized: *Delftia litopenaei* [42], *Delftia rhizosphaerae* [43] and *Delftia deserti* [44], though no genome sequences are yet available for these species.

Evolutionary relationships within the *Delftia* phylogeny formed two major clades: clade DA, *D. acidovorans *, that included *D. acidovorans* and 17 *Delftia* spp.; and clade DLT, *D. lacustris/D. tsuruhatensis*, that included *D. lacustris *, *D. tsuruhatensis *, 8 *Delftia* spp. and two isolates deposited in the National Center for Biotechnology Information (NCBI) database under the label of *D. acidovorans *. The DA clade comprised two sub-clades: DA1, which had the majority of isolates; and DA2, with a longer branch leading to *D. acidovorans* strains UBA3003, FDAARGOS_909 and new MAG-209, and *Delftia* sp. UME58 (Fig. 1). Within clade DLT, relationships between the three named species were polyphyletic, suggesting they, along with the *Delftia* spp. isolates, may be one species. The two *D. acidovorans* isolates in clade DLT have been previously noted to be more closely related to *D. tsuruhatensis* [39] and were likely misassigned due to being described before *D. lacustris* or *D. tsuruhatensis* were discovered. Estimates of population structure were consistent with three groups that corresponded to clades DA1, DA2 and DLT (Table S1).

**Fig. 2.**
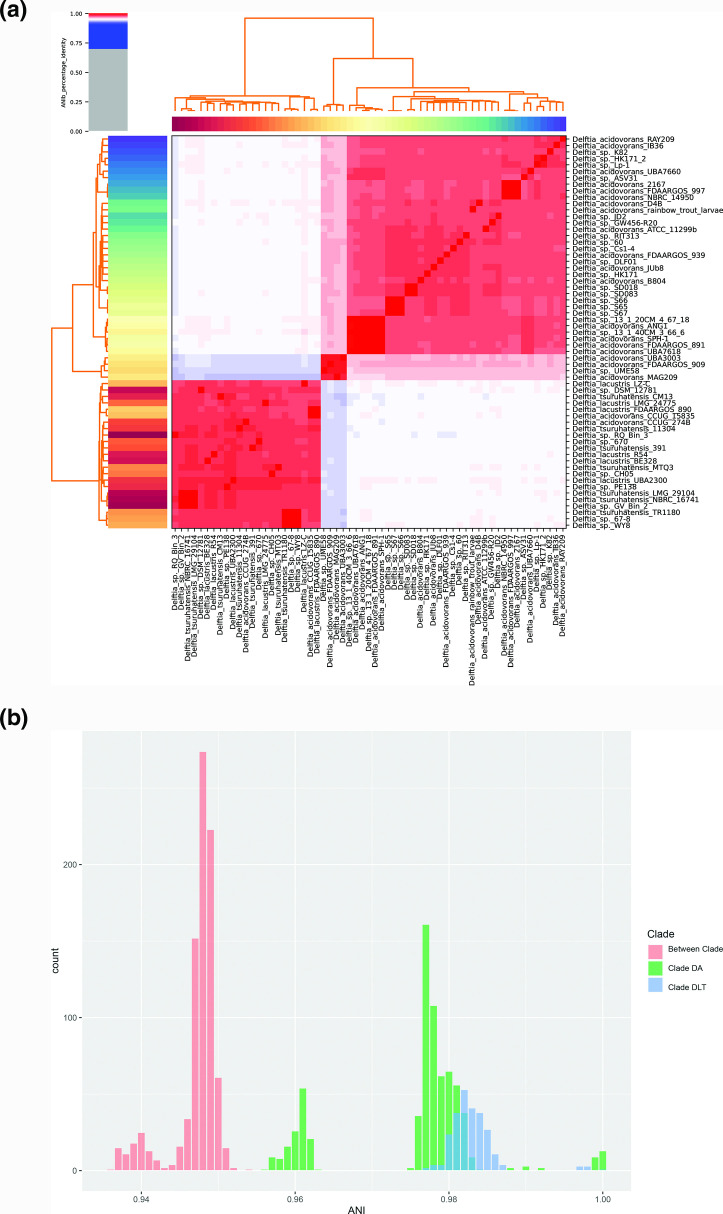
ANI estimates for 61 *Delftia* genomes. (a) A cluster diagram showing ANI values between each pairwise comparison. (**b**) Distribution of ANI values for between-clade and within-clade comparisons.

ANI values were consistent with the results from phylogenetic inference, showing three ANI clusters that corresponded to clades DA1, DA2 and DLT (Fig. 2a). The mean ANI for comparisons between clades DA and DLT was 94.7 %, indicating these clades represent different species based on the 95.0 % intraspecies ANI cut-off [40] (Fig. 2b). The mean ANI within clade DA was 97.5 %. Separating DA2 for comparison showed that the four genomes in clade DA2 were on average 96 % identical to members of the DA1 clade, and 94 % identical to members of the DLT clade, indicating DA2 may be forming a new species. Within the DLT clade, the mean ANI was 98.3%, a value consistent with *D. lacustris* and *D. tsuruhatensis* representing a single species.

### Core genomes and proteomes

*Delftia* spp. are known for their ecological versatility, which is reflected in their relatively large pan-genome sizes. The pan-genome size was 17 986 genes for clade DA and 18 987 genes for clade DLT. Gene accumulation curves indicated that the pan-genomes for both DA and DLT are open, as the total number of genes in each pan-genome was still increasing as additional genomes were included; further, estimates of the power law parameter gamma were greater than 0 (Fig. S1) [27]. Thus, more genes are expected to be discovered with additional sequencing of *Delftia* genomes.

The core-genome size was 2060 genes for clade DA and 2092 genes for clade DLT. These numbers of core genes are within the range of those from diverse bacterial species (746–3668 genes) [45], and consistent with the 2905 core-gene families previously found in 31 *Delftia* genomes (mean 2538 core genes with 31 genomes sampled in our dataset) [46].

Comparing gene content at the genus level indicated a *Delftia* pan-genome size of 28 430 genes, only 884 of which were found in all 61 genomes. Gene distributions closely matched the structure of the phylogeny, highlighting the existence of two main clades and a distinguishable DA2 clade (Fig. S2).

### *Delftia* ecological diversity

**Fig. 3.**
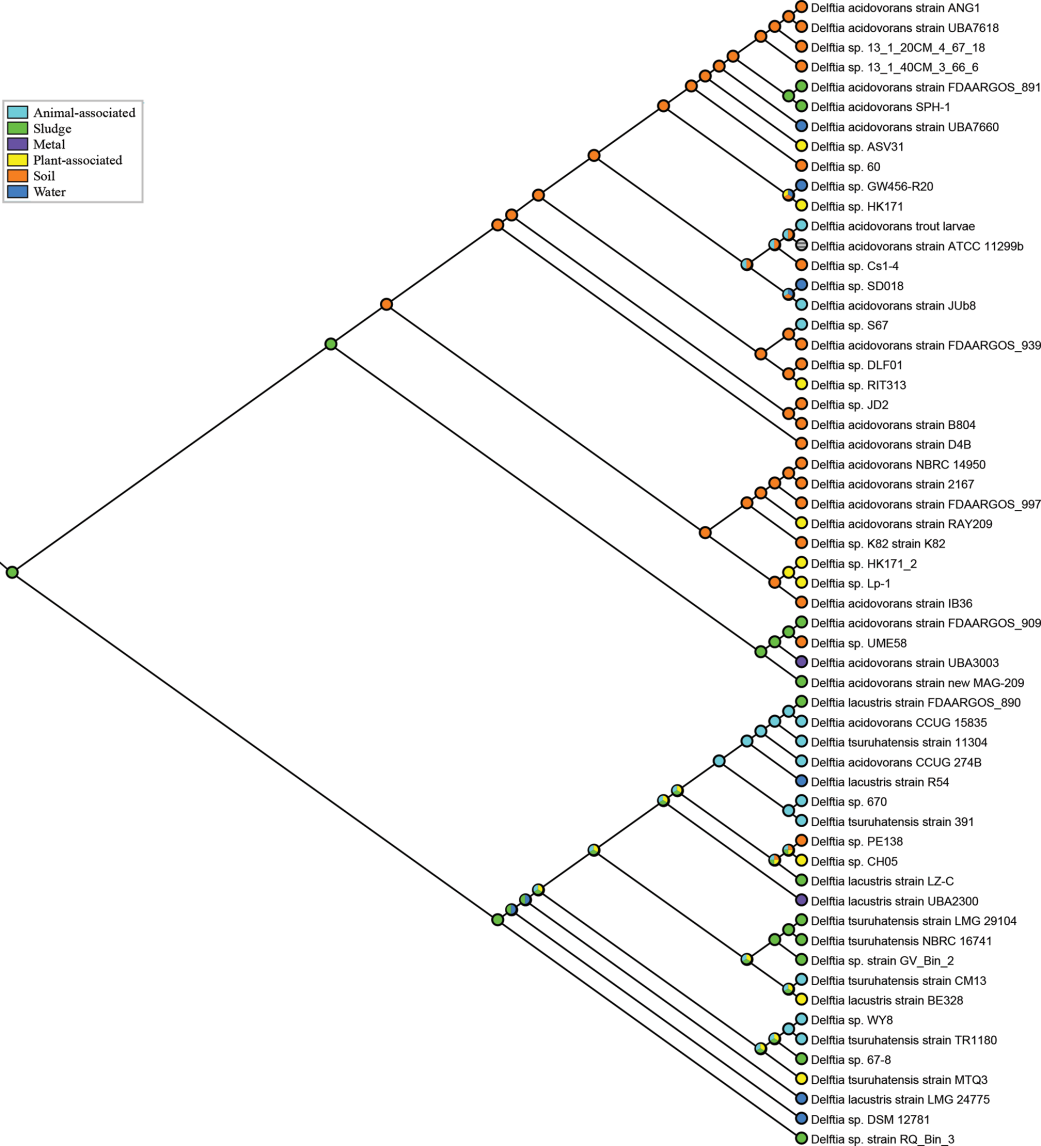
Parsimony reconstruction of ancestral habitats for *Delftia *. The phylogeny is the same as shown in Fig. 1. Circles indicate habitat of isolation for terminal branches and ancestral predictions for internal nodes.

Of the 35 isolates in clade DA, with known sources of isolation, 17 (49 %) were isolated from soil, 6 (17 %) from plants, 3 (9%) from water, 4 (11 %) from sludge, 1 (3 %) from metal and 3 (9%) from animals (1 fish, 1 insect, 1 nematode; Figs 1 and 3, Table S1). Of the 23 isolates in clade DLT, 1 (4 %) was isolated from soil, 3 (13 %) from plants, 3 (13 %) from water, 7 (30 %) from sludge, 1 (4%) from metal and 8 (35 %) from animals (7 humans, 1 krill). Overall, members of clade DA appeared to be more often soil-dwelling where they would interact with plants and other soil organisms (e.g. insects, worms), whereas members of clade DLT appear to be more often isolated from industrial-derived sludges or humans. Clade DA2 included isolates from sludge, soil and metal, indicating that their ecology has diverged from clade DA1 and is perhaps more similar to that of clade DLT, though additional isolates would be required to determine whether these trends are due to sampling artefacts.

Habitats for ancestral nodes were reconstructed using parsimony. This indicated that the ancestor of the DA1 clade was most likely soil-dwelling, whereas the ancestor of DLT clade inhabited sludge. The basal ancestor was inferred to be sludge-dwelling, with only clade DA1 evolving from a soil-dwelling ancestor. Both soil and sludge environments are habitats for other members of the *Comamonadaceae *, the family to which *Delftia* belongs, whose members are found in diverse environments [47].

Although clades DA and DLT appear to be ecologically specialized, their overall proteomes did not extensively differ. DA genomes encoded 27 proteins, including porins, transporters and enzymes, not found in clade DLT (Table S2). DLT genomes encoded 12 proteins not found in DA, including an ABC transporter efflux pump. No proteins were found to be specific to isolates from a particular habitat.

Separating the two clades within DA showed that clade DA2 encoded 206 proteins not found in clade DA1 (nor DLT). These included many transporters, transcriptional regulators and enzymes (Table S3). In contrast, clade DA1 encoded only 11 proteins not found in DA2 (nor DLT).

### Applied *Delftia* genomics

The applied microbiology of *Delftia* includes plant-growth promotion and bioremediation. Both of these applications depend on a bacterial strain possessing genes for specialized functions. As a crop inoculant, *Delftia* strains require the ability to colonize the rhizosphere and attach to roots, and may involve mobilization of nutrients for the plant host; at the same time, strains destined for environmental release will need to lack pathogenic potential. Similarly, using *Delftia* in mining and bioremediation benefits from a strain being able to engage in redox reactions with heavy metals. Here, we examine gene content predictions to support strain selection and engineering.

#### Plant root colonization

The genus *Delftia* has been associated with PGPR [16]. Root colonization is often associated with PGPR and has been studied in *D. acidovorans* RAY209 [48]. Of 50 RAY209 genes previously found to have altered transcription during colonization of canola or soybean roots [49], 17 were detected in all 61 *Delftia* genomes and 29 were detected in at least 60 genomes (Fig. S3). One gene that encoded a putative membrane transporter (RAY209 locus CHL79_06455) was present in all members of clade DA, except for *D. acidovorans* strain MAG-209 in clade DA2. This gene was present in only two isolates from clade DLT: *D. tsuruhatensis* strain MTQ3 (isolated from plants) and *Delftia* sp. strain RQ_Bin_3 (isolated from sludge) (Figs 1 and S3). That nearly all of the genes whose expression increased during root colonization were found throughout members of the genus *Delftia *, including those strains that were not isolated from plants, indicates that the genetic potential for plant root colonization is likely ancestral and widespread in *Delftia *.

#### Metal metabolism

*Delftia* has been shown to use a secreted metabolite, delftibactin, to mineralize gold, a trait for detoxifying metals that is of industrial interest. The entire NRPS/PKS gene cluster whose products synthesize delftibactin, named the *del* cluster (Daci_4 753–Daci_4 759) [20], was encoded by 40 of the 61 *Delftia* genomes. *D. acidovorans* strain UBA7660 only had the *delH* gene (Daci_4753), whereas two genes of the core *del* locus, *delG* (Daci_4754) and *delF* (Daci_4755), were missing from 5 and 17 strains, respectively. A *delG* deletion mutant is unable to produce functional delftibactin [20]. Absent from 19 strains was the gene immediately upstream of the core *del* locus (CHL79_01015 in RAY209) that encodes FvpA, a TonB-dependent outer membrane receptor for delftibactin. Taken together, these results suggest that 21 of the 38 strains in clade DA (including all 4 strains in DA2), and 3 of the 23 strains in clade DLT, may not produce functional delftibactin, though additional phenotypic tests are required for conclusive evidence.

Encoded adjacent to the *del* cluster was a set of genes that are predicted to encode a CzcCBA-like heavy metal efflux system, a member of the resistance-nodulation-cell division (RND) protein family, that has been shown to confer resistance to Co, Zn, Cd and Ni [50]. These genes were all present in 37 of 38 clade DA strains and 22 of 23 clade DLT strains. Only *Delftia* strain 13_1_40 CM_3_66_6 (isolated from soil) and *Delftia* strain RQ_Bin_3 (isolated from sludge) lacked these genes.

#### Infectious potential and associations with humans

Several isolates in clade DLT were isolated from humans in the clinical environment. Indeed, *D. tsuruhatensis* is considered to be an emerging human pathogen, and a recent analysis of 15 strains showed their pan-genome encodes numerous putative virulence genes [22, 51]. No proteins were identified to be specific to the human isolates. Two isolates from individuals admitted to a hospital in China, *Delftia* sp. 670 [52] and *D. tsuruhatensis* strain 391, were each other’s closest relative and had more evolutionary change (long branch) compared to neighbouring lineages (Fig. 1). These two isolates encoded 74 proteins not found in any of the other 59 *Delftia* genomes (Table S4). These proteins included several RND efflux pumps and siderophore biosynthesis proteins, functions that contribute to host colonization in other bacteria. Moreover, *Delftia* sp. 670 and *D. tsuruhatensis* strain 391 had 19 and 20 % of their genes, respectively, predicted to be pseudogenes, compared to 0 or 1 % for other *Delftia* genomes. However, *Delftia* sp. 670 and *D. tsuruhatensis* strain 391 encoded the lowest percentage of virulence factors, 10.5 and 10.3 %, compared to the other 59 genomes that had between 12.2 and 14.7 % of genes predicted to be virulence factors (Table S1). The increased amount of genetic change in these two isolates compared to other *Delftia* could reflect genetic drift and gene decay, processes that accelerate evolution of generalist bacteria after colonization of the stable habitats on and in a human body [53].

### Conclusion

Results indicate that *D. acidovorans* strains preferentially inhabit soil and plant rhizospheres, whereas *D. lacustris* and *D. tsuruhatensis* strains inhabit sludge environments. Despite the difference in habitat preferences, *Delftia* bear the hallmarks of generalists as many strains encode proteins that would enable them to thrive in many environments. The genome analysis has also revealed that opportunistic pathogens and animal colonizers arise in both major clades, and strains within the clade DLT appear to be particularly capable of causing opportunistic infection.

## Supplementary Data

Supplementary material 1

Supplementary material 2
