# Supplementary material for: Phylogenomic analysis of the genus Delftia reveals distinct major lineages with ecological specializations
Source: Microb Genom. 2022 Sep 15;8(9):mgen000864. doi: 10.1099/mgen.0.000864 (PMC9676026; doi:10.1099/mgen.0.000864)
Supplement: Supplementary material 1 [file mgen-8-864-s001.pdf]

Supplementary Figure S1. Number of new genes in the pangenomes with the inclusion of each additional genome for (A) *D. acidovorans* (Clade DA) and (B) *D. lacustris* and *D. tsuruhatensis* (Clade DLT). Gamma parameter values from power law regressions were 0.319 for Clade DA and 0.395 for Clade DLT.

Supplementary Figure S2. Presence of genes within *Delftia* genomes. Each blue tick represents one gene.

Supplementary Figure S3. Extended version of Figure 1 showing distributions of genes encoding plant root colonization and metal metabolism genes in *Delftia*. Each colored box indicates the presence of the gene listed at the top (gene numbers are according to *D. acidovorans* strain RAY209). Grey boxes indicate absence.

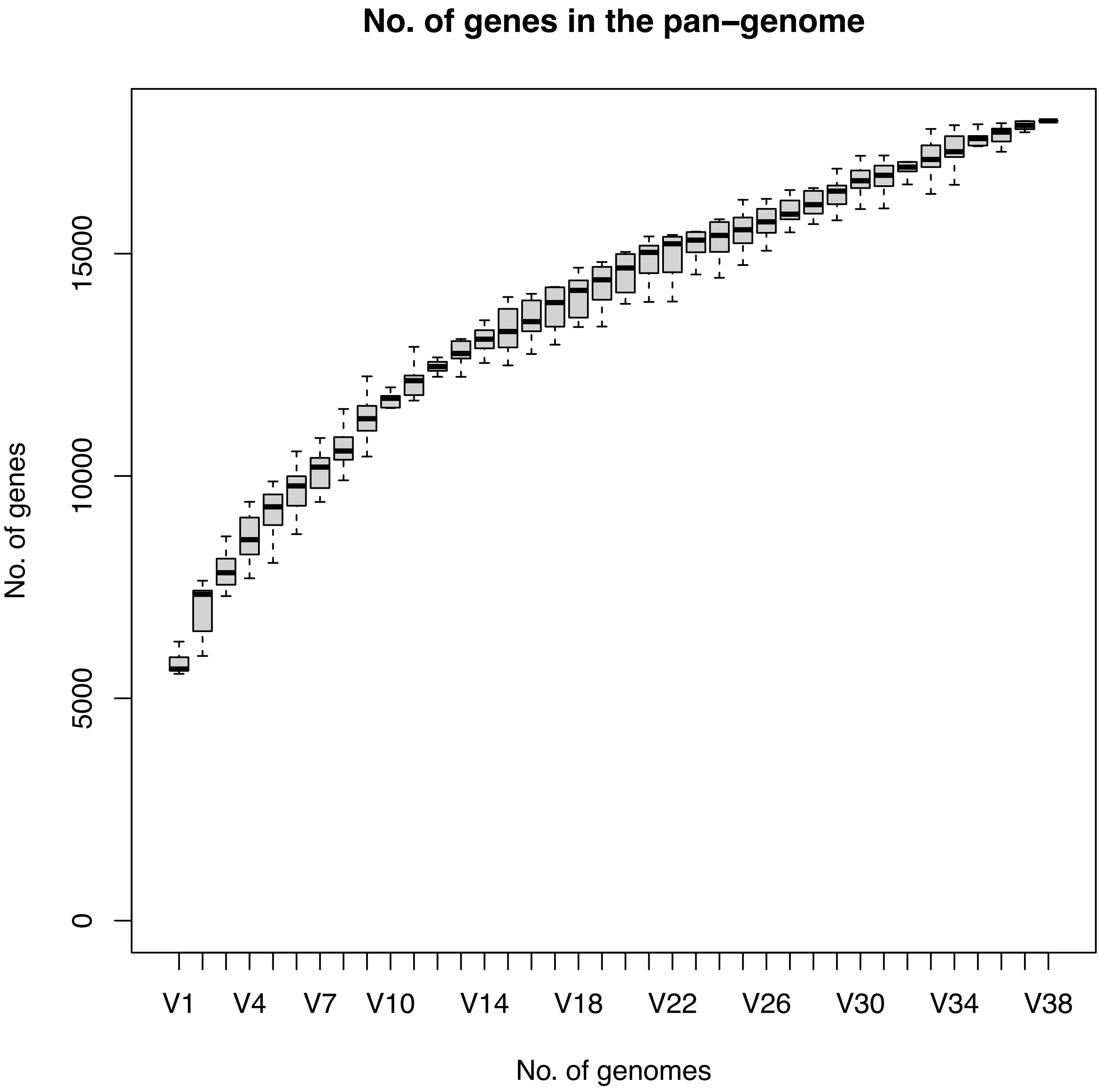

No. of genes in the pan−genome
No. of genes
0
5000
10000
15000
No. of genomes
V1
V4
V7
V10
V14
V18
V22
V26
V30
V34
V38

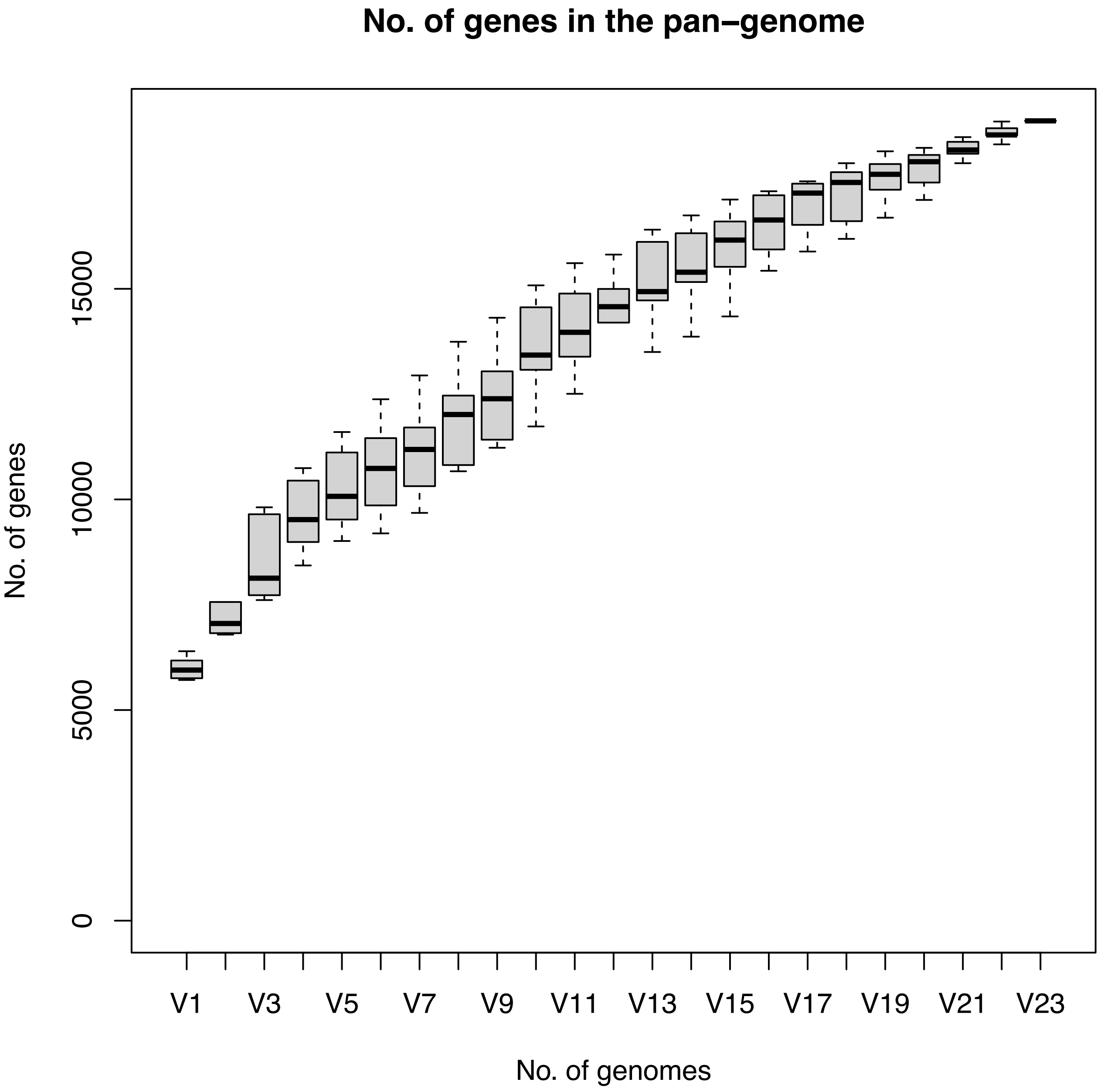

No. of genes in the pan−genome
No. of genes
0
5000
10000
15000
No. of genomes
V1
V3
V5
V7
V9
V11
V13
V15
V17
V19
V21
V23

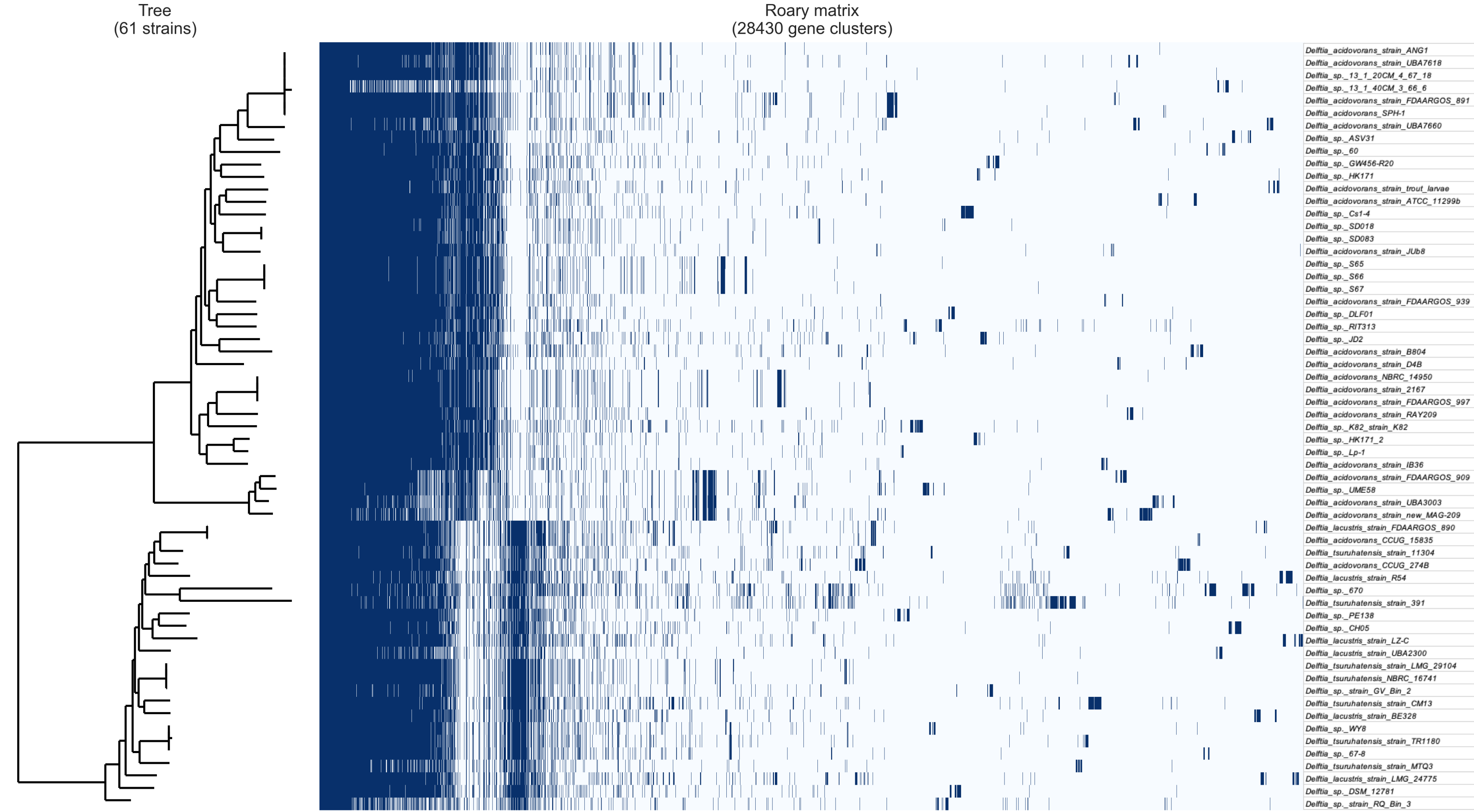
Tree
(61 strains)
Roary matrix
(28430 gene clusters)
Delftia_acidovorans_strain_ANG1
Delftia_acidovorans_strain_UBA7618
Delftia_sp._13_1_20CM_4_67_18
Delftia_sp._13_1_40CM_3_66_6
Delftia_acidovorans_strain_FDAARGOS_891
Delftia_acidovorans_SPH-1
Delftia_acidovorans_strain_UBA7660
Delftia_sp._ASV31
Delftia_sp._60
Delftia_sp._GW456-R20
Delftia_sp._HK171
Delftia_acidovorans_strain_trout_larvae
Delftia_acidovorans_strain_ATCC_11299b
Delftia_sp._Cs1-4
Delftia_sp._SD018
Delftia_sp._SD083
Delftia_acidovorans_strain_JUb8
Delftia_sp._S65
Delftia_sp._S66
Delftia_sp._S67
Delftia_acidovorans_strain_FDAARGOS_939
Delftia_sp._DLF01
Delftia_sp._RIT313
Delftia_sp._JD2
Delftia_acidovorans_strain_B804
Delftia_acidovorans_strain_D4B
Delftia_acidovorans_NBRC_14950
Delftia_acidovorans_strain_2167
Delftia_acidovorans_strain_FDAARGOS_997
Delftia_acidovorans_strain_RAY209
Delftia_sp._K82_strain_K82
Delftia_sp._HK171_2
Delftia_sp._Lp-1
Delftia_acidovorans_strain_IB36
Delftia_acidovorans_strain_FDAARGOS_909
Delftia_sp._UME58
Delftia_acidovorans_strain_UBA3003
Delftia_acidovorans_strain_new_MAG-209
Delftia_lacustris_strain_FDAARGOS_890
Delftia_acidovorans_CCUG_15835
Delftia_tsuruhatensis_strain_11304
Delftia_acidovorans_CCUG_274B
Delftia_lacustris_strain_R54
Delftia_sp._670
Delftia_tsuruhatensis_strain_391
Delftia_sp._PE138
Delftia_sp._CH05
Delftia_lacustris_strain_LZ-C
Delftia_lacustris_strain_UBA2300
Delftia_tsuruhatensis_strain_LMG_29104
Delftia_tsuruhatensis_NBRC_16741
Delftia_sp._strain_GV_Bin_2
Delftia_tsuruhatensis_strain_CM13
Delftia_lacustris_strain_BE328
Delftia_sp._WY8
Delftia_tsuruhatensis_strain_TR1180
Delftia_sp._67-8
Delftia_tsuruhatensis_strain_MTQ3
Delftia_lacustris_strain_LMG_24775
Delftia_sp._DSM_12781
Delftia_sp._strain_RQ_Bin_3

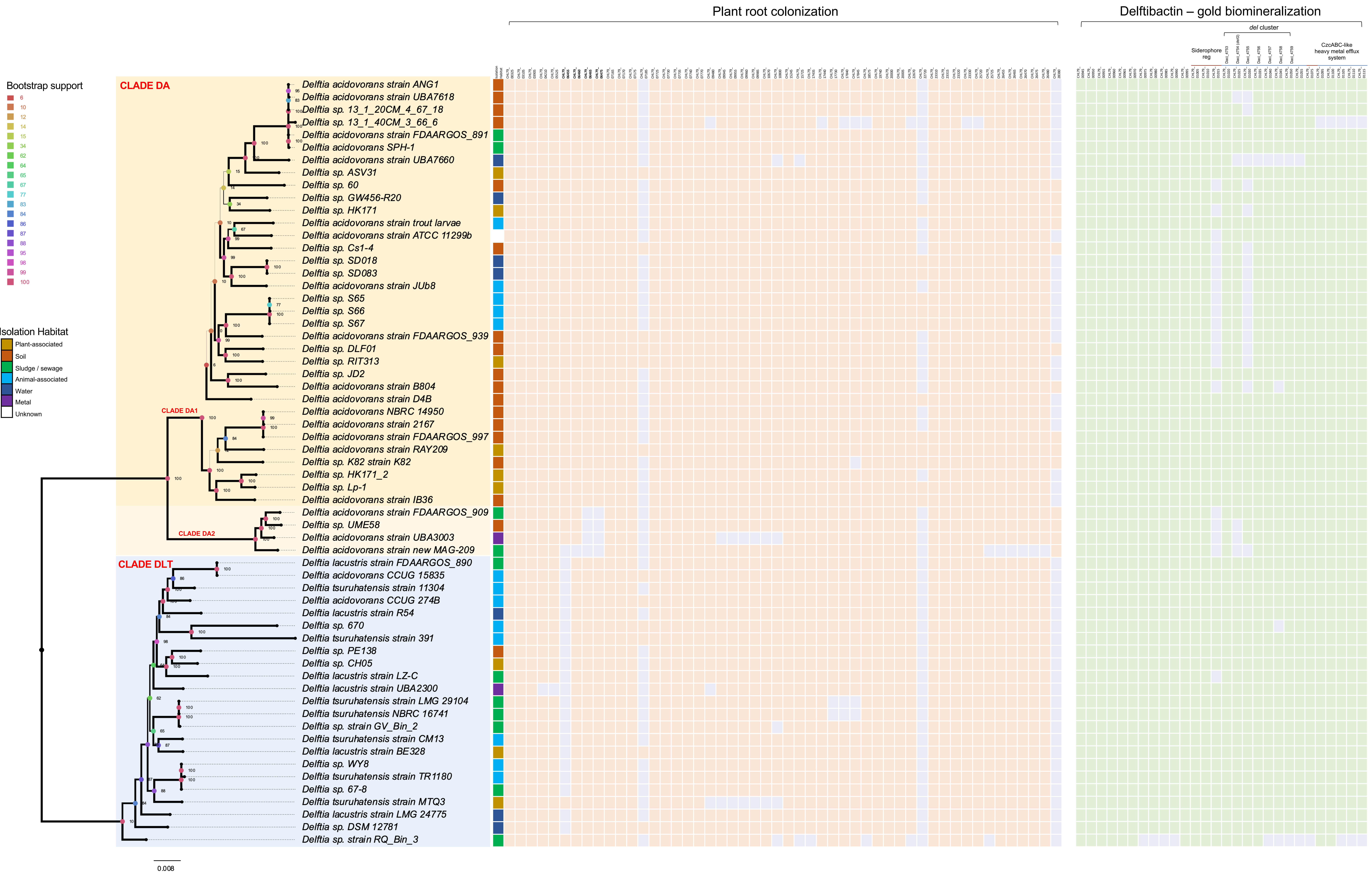
Plant root colonization
Delftibactin – gold biomineralization
del cluster
Siderophore reg
CzcABC-like heavy metal efflux system
Bootstrap support
6
10
12
14
15
34
62
64
65
67
77
83
84
86
87
88
95
98
99
100
Isolation Habitat
Plant-associated
Soil
Sludge / sewage
Animal-associated
Water
Metal
Unknown
CLADE DA
CLADE DA1
CLADE DA2
CLADE DLT
Delftia acidovorans strain ANG1
Delftia acidovorans strain UBA7618
Delftia sp. 13_1_20CM_4_67_18
Delftia sp. 13_1_40CM_3_66_6
Delftia acidovorans strain FDAARGOS_891
Delftia acidovorans SPH-1
Delftia acidovorans strain UBA7660
Delftia sp. ASV31
Delftia sp. 60
Delftia sp. GW456-R20
Delftia sp. HK171
Delftia acidovorans strain trout larvae
Delftia acidovorans strain ATCC 11299b
Delftia sp. Cs1-4
Delftia sp. SD018
Delftia sp. SD083
Delftia acidovorans strain JUb8
Delftia sp. S65
Delftia sp. S66
Delftia sp. S67
Delftia acidovorans strain FDAARGOS_939
Delftia sp. DLF01
Delftia sp. RIT313
Delftia sp. JD2
Delftia acidovorans strain B804
Delftia acidovorans strain D4B
Delftia acidovorans NBRC 14950
Delftia acidovorans strain 2167
Delftia acidovorans strain FDAARGOS_997
Delftia acidovorans strain RAY209
Delftia sp. K82 strain K82
Delftia sp. HK171_2
Delftia sp. Lp-1
Delftia acidovorans strain IB36
Delftia acidovorans strain FDAARGOS_909
Delftia sp. UME58
Delftia acidovorans strain UBA3003
Delftia acidovorans strain new MAG-209
Delftia lacustris strain FDAARGOS_890
Delftia acidovorans CCUG 15835
Delftia tsuruhatensis strain 11304
Delftia acidovorans CCUG 274B
Delftia lacustris strain R54
Delftia sp. 670
Delftia tsuruhatensis strain 391
Delftia sp. PE138
Delftia sp. CH05
Delftia lacustris strain LZ-C
Delftia lacustris strain UBA2300
Delftia tsuruhatensis strain LMG 29104
Delftia tsuruhatensis NBRC 16741
Delftia sp. strain GV_Bin_2
Delftia tsuruhatensis strain CM13
Delftia lacustris strain BE328
Delftia sp. WY8
Delftia tsuruhatensis strain TR1180
Delftia sp. 67-8
Delftia tsuruhatensis strain MTQ3
Delftia lacustris strain LMG 24775
Delftia sp. DSM 12781
Delftia sp. strain RQ_Bin_3
0.008
